# Patient Factors Predicting Success in Lateral Graft Tympanoplasty

**DOI:** 10.1097/ONO.0000000000000015

**Published:** 2022-07-20

**Authors:** Zoha K. Momin, Kristen L. Yancey, Tanner J. Mitton, Joe Walter Kutz

**Affiliations:** 1 Department of Otolaryngology-Head and Neck Surgery, University of Texas Southwestern Medical Center, Dallas, TX.

**Keywords:** Advanced age, Diabetes, Lateral graft tympanoplasty, Smoking, Tympanoplasty

## Abstract

**Objective:**

To compare the effects of preoperative medical comorbidities and operative findings on the success of lateral graft tympanoplasty.

**Study Design:**

Retrospective chart review.

**Setting:**

Tertiary medical center.

**Patients:**

Ninety-six patients undergoing lateral graft tympanoplasty from December 2008 to November 2020 with at least 2 months follow-up were included. Patient demographics, comorbidities including smoking status, intraoperative findings, and healing, and hearing outcomes were recorded.

**Interventions:**

Lateral graft tympanoplasty.

**Main Outcome Measures:**

The primary outcome was perforation closure. Secondary outcomes were postoperative complications and change in air-bone gap (ABG).

**Results:**

Ninety-nine ears (mean age 40.94 ± 18.44 years) were included. Tympanic membrane perforation closure was achieved in 92 (92.9%) ears. Perforation closure was not associated with diabetes (*P* > 0.99), smoking (*P* > 0.99), or the presence of cholesteatoma at the time of lateral graft tympanoplasty (*P* = 0.10). Increased age (odds ratio [OR] = 1.04, *P* = 0.31) was also not correlated with tympanic membrane closure rate. An absent malleus resulted in a higher rate of lateralization (31.3% versus 2.1%; OR = 18.41, 95% confidence interval [CI] = 3.09-95.95, *P* = 0.001) but not blunting (12.5% versus 4.8%; OR = 0.24, 95% CI = 0.49-12.93, *P* = 0.24). The mean ABG improved 6.82 ± 11.33 dB (*P* < 0.01). History of prior tympanoplasty was associated with smaller ABG improvement following surgery (ß = 4.038, *R*^2^ = 0.262, *P* = 0.04) but not perforation closure (OR = 3.25, 95% CI = 0.63-16.81, *P* = 0.24).

**Conclusions:**

Diabetes, active smoking, and advancing age were not associated with adverse healing in patients undergoing lateral graft tympanoplasty. Lateralization was more common with an absent malleus.

Tympanic membrane (TM) perforations may occur in the setting of acute or chronic ear disease, trauma, and following surgery. A successful tympanoplasty aims to create a dry, safe ear and correct conductive hearing loss. Perforation closure may also help decrease otorrhea in hearing aid users (1). First described by Sheehy and Glasscock (2) in the 1960s, lateral graft or overlay tympanoplasty involves graft placement lateral to the annulus and medial to the malleus. Lateral graft tympanoplasty can be a useful treatment modality for total, subtotal, and anterior perforations, as well as select cases where maximal middle ear exposure may be required (3–5). The operation requires removal of canal epithelium and the remnant TM (2,6). Since the TM and external auditory canal (EAC) skin are replaced, a lateral graft tympanoplasty takes longer to heal than an underlay tympanoplasty (7). Potential pitfalls of the technique include failure to remove all squamous epithelium resulting in an iatrogenic cholesteatoma or lateralization of the graft with blunting of the anterior sulcus with resulting hearing loss (4).

While previous studies comparing lateral graft to other tympanoplasty techniques have found similar rates of perforation closure and improved hearing (8,9), certain patient populations may be at risk for compromised healing, including active smokers (10–15), diabetics (14), and those of advanced age (16–19). The surgical outcomes following lateral graft tympanoplasty for these at-risk patient groups are not well-established (3,4,14,20). This study evaluates the potential association of patient-specific factors including diabetes, smoking, age, and prior tympanoplasty with perforation closure rate and subsequent hearing outcomes following lateral graft tympanoplasty by a single surgeon.

## MATERIALS AND METHODS

This study was approved by the Institutional Review Board at (STU 012013-017). All patient data were collected and securely stored in Research electronic data capture (21,22).

### Patient Population

A retrospective chart review was performed for patients that underwent a lateral graft tympanoplasty from December 2008 to November 2020 by a single surgeon. Patients were excluded if they had less than 2 months of postoperative follow-up. In general, the senior author follows patients for 1-year postoperatively, barring ongoing or recurrent disease. Patient demographics, comorbidities of interest (smoking, diabetes), and operative findings were reviewed. Diabetes diagnosis and smoking status were obtained from the preoperative anesthesia evaluation and problem list within the electronic medical record.

### Surgical Details

All surgeries were performed with an operating microscope via a postauricular approach. A temporalis graft is harvested for the reconstruction. Postauricular vascular strip incisions are then made, the remaining EAC skin is removed, and a canaloplasty is performed to remove the acute anterior angle of the EAC and improve visualization of the bony annulus. Residual TM, including the fibrous annulus, is removed, and the anterior bony annulus is deepened with a 2 mm diamond burr.

After addressing any middle ear disease that may be present, the reconstruction is performed, typically with a temporalis graft. Depending on the clinical scenario (eg, recurrent perforations status post multiple attempted repairs [N = 18 of 34 ears]), a composite 8-mm shield graft of tragal cartilage and perichondrium may be used to bolster the fascia. A 3-mm notch is made in the temporalis instead of fascia to accommodate the manubrium, and the graft is laid over the middle ear space and onto the bony annulus, while also sliding under the malleus. If the malleus is absent, an anterior tab is created in the graft and placed under the bony annulus near the region of the protympanum and eustachian tube. When an ossicular reconstruction is indicated, tragal cartilage is also used to cover the prosthesis. After trimming frayed edges, the anterior canal skin is returned to the canal, with approximately 2-3 mm overlying the TM graft. Gelatin sponge soaked in fluoroquinolone antibiotic is then tightly packed into the anterior sulcus to prevent blunting. The vascular strip is returned to its anatomic position and the remainder of the canal is then packed, concluding the procedure after standard closure of the periosteal and skin incisions.

### Surgical Outcomes

Perforation size (expressed as a percentage of the pars tensa portion of the TM), location, and presence of coexisting cholesteatoma were recorded. Surgical success was defined as perforation closure with a healthy appearing TM. Wound healing issues, including significant postoperative myringitis requiring topical treatment, graft lateralization, blunting of the anterior sulcus, and EAC cholesteatomas larger than small, isolated foci were tracked.

### Audiologic Outcomes

Preoperative and postoperative audiometric data were reviewed. Patients undergoing surgery for medial canal fibrosis were excluded from the hearing analysis. The most recently recorded postoperative audiogram was used for each patient. In patients that underwent revision or scheduled second-look surgeries, the most recent postoperative audiogram following their last surgery was analyzed. Air-bone gaps (ABGs) were derived from the difference between the air and bone conduction thresholds, averaged across 500, 1000, 2000, and 3000 Hz. Pure-tone averages (PTAs) were obtained by averaging air conduction thresholds across 500, 1000, 2000, and 3000 Hz. For both ABGs and PTAs, if 3000 Hz was not available, the mean of 2000 and 4000 Hz was used. Preoperative to postoperative PTAs and word recognition scores are presented per American Academy of Otolaryngology - Head and Neck Surgery guidelines 2012 (23).

### Statistical Analysis

The main endpoint was TM perforation closure. Secondary endpoints included postoperative healing complications and hearing outcomes. Patient age was treated as a continuous variable. Univariate logistic regression was used to evaluate perforation closure as related to age as well as postoperative healing complications by age. Associations between malleus status and postoperative healing complications were evaluated with Fisher exact test. Fisher exact test was also used to evaluate the relationships between perforation closure and smoking and diabetes status, cholesteatoma, and prior tympanoplasty.

Changes in preoperative and postoperative ABG were compared with paired *t* tests. Linear regression analysis was performed to evaluate the potential effect of diabetes, smoking status, age, cholesteatoma, and prior tympanoplasty on postoperative ABG, controlling for preoperative hearing. Hearing outcomes (postoperative ABG) were further assessed by the type of reconstruction material used as well as the status of the ossicular chain by Kruskal-Wallis test. Patients were stratified into 5 groups: 1) fascia-only graft, 2) perichondrium-cartilage composite graft, 3) limited ossicular mobility (lateral chain or stapes), and ossicular discontinuity that did 4) or did not 5) undergo ossicular chain reconstruction (OCR).

Statistical analyses were performed using Stata 16.1 2019 (STATACorp LLC, College Station, TX). Statistical significance was set at *P* < 0.05.

## RESULTS

Ninety-six patients (99 ears) were included for review, with a mean age and SD of 40.94 ± 18.44 years (range, 15-85 years) (Table 1). Mean follow-up duration and SD was 33.34 ± 30.81 months. A concurrent mastoidectomy was performed in 26 (26.3%) ears, and an OCR was done in 18 (18.2%) cases. Six patients were previously diagnosed with diabetes, and eleven patients were active smokers at the time of surgery. All diabetic patients were characterized as well-controlled in their preoperative evaluation visits with anesthesia. Median perforation size was 50% (range, 10%-100%).

**TABLE 1.** Summary characteristics of patients undergoing lateral graft tympanoplasty

Patient characteristics	No. ears (%)
Number of ears	99
Number of patients	96
Age at surgery (years) (mean ± SD)	40.94 ± 18.44
Follow-up time (months) (mean ± SD)	33.34 ± 30.81
Female	51 (51.5)
Prior tympanoplasty	45 (45.5)
Right ear	50 (50.5)
Diabetes	6 (6.1)
Active smoking	11 (11.1)
Intraoperative cholesteatoma	29 (29.3)
Perforation size	
<25%	6 (7.1)
25%-49%	31 (36.5)
50%-74%	15 (17.6)
>75%	33 (38.8)

### Perforation Closure and Complications

Overall, perforations were closed in the majority of ears (N = 92, 92.9%) without revision surgery. Likelihood of closure was not associated with age (odds ratio [OR] = 1.04, *P* = 0.31) on logistic regression analysis. Diabetes (*P* > 0.99) and smoking (*P* > 0.99) did not appear to impact perforation closure by Fisher exact test (Table 2). The presence of cholesteatoma at the time of lateral graft tympanoplasty (*P* = 0.10) and history of prior tympanoplasty (*P* = 0.24) did not increase the odds of having a residual perforation.

**TABLE 2.** Residual perforation following lateral graft tympanoplasty by patient factor analyzed by Fisher exact test

Prognostic factor	OR (95% CI)	Residual perforation	*P*
		No. ears (%)	
Diabetes	0.00 (0.00-8.35)	0 (0)	>0.99
Smoker	0.00 (0.00-5.41)	0 (0)	>0.99
Intraoperative cholesteatoma	0.00 (0.00-1.33)	0 (0)	0.10
Prior tympanoplasty	3.25 (0.63-16.81)	5 (11.1)	0.24

Overall, tympanic membrane perforations healed in 92 patients (92.9%) following initial surgery.

CI indicates confidence interval; OR, odds ratio.

Seven ears (7.1%) underwent treatment for myringitis with topical antibiotic and steroid drops. There was no association between age and myringitis (OR = 0.98, *P* = 0.42) by univariate logistic regression. Two ears developed EAC cholesteatoma. Additionally, advancing age did not appear to influence lateralization (N = 7 ears, 7.1%; OR = 0.96, *P* = 0.07), or blunting (N = 6 ears, 6.1%; OR = 1.03, *P* = 0.11). Of the 7 ears that developed postoperative lateralization, the malleus was absent (N = 4) or partially eroded (N = 1) at the time of lateral grafting in 5 cases (71.4%). Ears with an abnormal malleus (N = 16) had 18 times the odds of developing lateralization (OR = 18.41, 95% confidence interval [CI] = 3.09-95.95, *P* = 0.001). The incidence of blunting was also higher in ears without a malleus at 12.5% (N = 2), compared with 4.8% (N = 4) when the malleus was present, but this did not reach the level of statistical significance (OR = 2.82, 95% CI = 0.49-12.93, *P* = 0.25).

### Additional Interventions

Revision surgery following initial lateral graft tympanoplasty was required in 9 (9.1%) ears to address persistent perforation (N = 6), cholesteatoma (N = 2), or an extruded prosthesis (N = 1). An additional patient with persistent perforation opted against surgical management. One perforation persisted despite revision surgery, and the patient declined further intervention.

Following tympanoplasty for extensive chronic ear disease, a planned second-look surgery was performed in eleven (11.1%) ears. During the second look, OCR was performed in 9 (81.8%) ears, and recurrent cholesteatoma was encountered and removed in 2 (18.2%) ears. Two (18.2%) cases then underwent an additional, third surgery, either for revision ossicular reconstruction (N = 1) or cholesteatoma (N = 1) that developed following the second look. While the patient with recurrent cholesteatoma was an active smoker, both cases involved patients <60 years old.

### Audiologic Outcomes

**FIG. 1.**
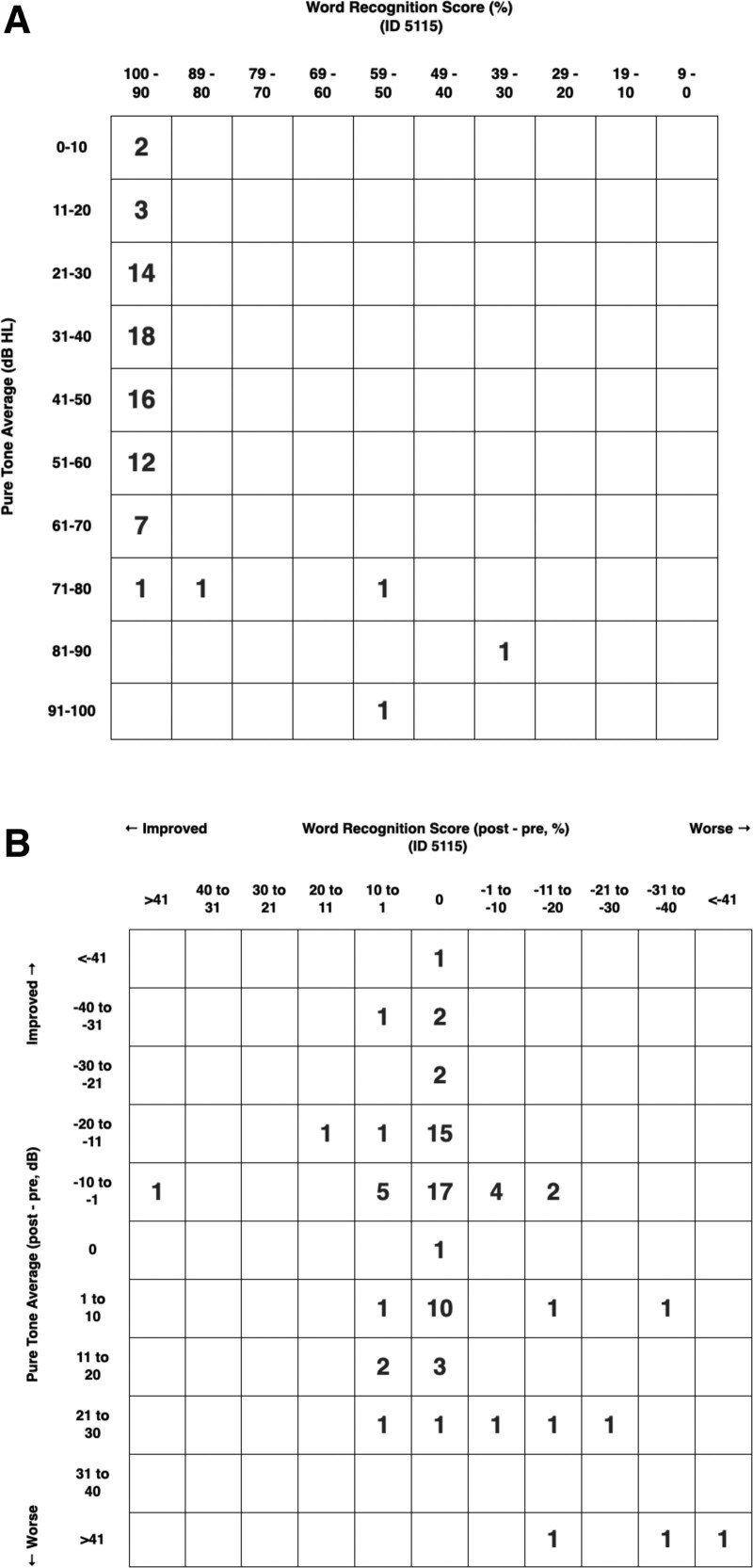
Standardized pre- and postoperative hearing scattergrams. *A*, Preoperative scattergram of PTAs (y-axis) and WRS (x-axis) (N = 79 ears). *B*, Preoperative to postoperative scattergram of hearing results for the same sample. Ears with improved postoperative hearing as represented by PTA, WRS, or both are shown in the left upper quadrant. Five ears were excluded as WRS testing was not performed. dB HL indicates hearing loss in decibels; ID, identification; PTA, pure-tone average; WRS, word recognition scores.

Preoperative and postoperative audiometric data were available for 84 ears. Patients had improvement in conductive hearing loss following surgery, with a statistically significant decrease in preoperative ABG of 28.18 ± 11.52 dB to 21.36 ± 10.52 dB (*P* < 0.01). Postoperative ABG fell within the range of 0-10 dB in 10.7% of ears (N = 9), 11-20 dB in 42.9% (N = 36), 21-30 dB in 25.0% (N = 21) and was >30 dB in 21.4% (N = 18). Preoperative PTA decreased from 44.05 ± 17.92 dB (range, 7.5-105 dB) to 41.57 ± 22.96 dB (range, 11.25-111.25 dB) postoperatively (N = 79 ears, Fig. 1). Mean time to postoperative audiogram following initial surgery was 24.98 ± 25.74 months (range, 2-115 months).

**FIG. 2.**
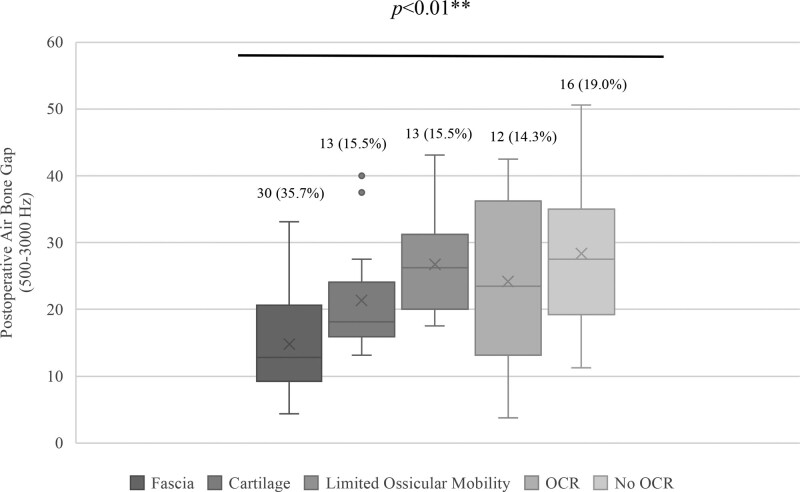
Postoperative ABG by graft material or ossicular chain status. Patient ears were stratified by 1) fascia-only graft, 2) perichondrium-cartilage composite graft, 3) limited ossicular mobility (lateral chain or stapes), and ossicular discontinuity that 4) did or 5) did not undergo OCR. The boxplots display median residual ABG and interquartile ranges for each group, with mean ABG represented by an “X.” Dots indicate outliers. The number of ears (%) in each category are shown above each boxplot. Asterisks indicate statistical significance by Kruskal-Wallis test. ABG indicates air-bone gap; OCR, ossicular chain reconstruction.

Hearing outcomes were affected by the type of reconstruction materials used and the status of the ossicular chain (Fig. 2). Ears with limited ossicular mobility (N = 13, 15.5%) or discontinuity that did not undergo OCR (N = 12, 14.3%) had larger ABGs at 26.73 ± 8.18 dB and 28.33 ± 10.91 dB, respectively, compared with those that underwent OCR (N = 16, 19.0%, ABG = 24.18 ± 12.48 dB; *P* < 0.01). Patients with intact and mobile ossicular chains were subdivided by graft material, with larger postoperative ABGs occurring in those ears reconstructed with perichondrium-cartilage composite grafts (N = 13, 15.5%, ABG = 21.25 ± 8.52 dB) compared with fascia-only (N = 30, 35.7%, ABG = 14.77 ± 7.12 dB; *P* < 0.01). Of note, 18 patients with composite grafts had also undergone prior tympanoplasty.

Controlling for preoperative hearing, linear regression found a history of prior tympanoplasty was associated with smaller improvements in ABG (ß = 4.038, *R*^2^ = 0.262, *P* = 0.04). In ears undergoing revision tympanoplasty (N = 39), ABG improved by 5.19 ± 11.73 dB compared with 8.22 ± 10.90 dB in ears without prior tympanoplasty (N = 45; *P* = 0.22). Other patient factors such as smoking, comorbid diabetes, advancing age or intraoperative cholesteatoma, did not significantly correlate with postoperative changes in ABG.

## DISCUSSION

Lateral graft tympanoplasty has been associated with prolonged healing relative to other techniques (7), and healing may be further compromised in patients with diabetes (14), ongoing smoking histories (10–15), and older age (16–19). However, our study did not find these patient groups to experience worse outcomes with respect to perforation closure or hearing outcomes.

To our knowledge, no other study has specifically evaluated the association between smoking or diabetes and healing or hearing outcomes following lateral graft tympanoplasty. Our results are consistent with those of medial (underlay), over-underlay, and inlay-graft techniques in diabetic and smoking populations (11–18,24–26). Odat et al. (27) found no associations between smoking, age, sex, or perforation type, and outcomes, but they did not distinguish between medial or lateral graft tympanoplasties. Similarly, other reviews of medial grafts (12,24,25,28) have not found worse TM healing or audiologic outcomes among smokers. However, these findings are not universal, with some reports noting smoking appeared to compromise graft uptake and negatively impacted hearing gains following tympanoplasty (13,15,16,26). In patients with comorbid diabetes, Lin et al. (14) found no significant association with surgical success or hearing improvement following inlay cartilage tympanoplasty (N = 6). For medial graft tympanoplasty, Jolink et al. (18) also encountered similar rates of TM healing and complications in patients with and without multimorbidity (defined by 3 or more chronic diseases, including diabetes).

In our series, age was not correlated with hearing outcomes. In contrast, other studies have reported decreased postoperative hearing improvement in older patients (11,29,30). Across various tympanoplasty techniques, this could be attributed to worse baseline hearing in aging patients. Regarding healing and age, our findings align with prior studies on lateral (3,4) and other tympanoplasty techniques (11,13,16–19,29) that have not found significant differences in outcomes as patients age. Instead, healing outcomes were influenced by the status of the malleus, with lateralization occurring in 31.3% of ears lacking a malleus compared with 2.1% cases where it was intact (*P* = 0.001). The manubrium is important to prevent lateralization of the neotympanum by serving as an anchor for the graft. One strategy to account for an absent malleus is to create a leading tab in the graft and place it under the anterior, superior bony annulus and into the protympanum to prevent blunting and lateralization of the TM. Despite such techniques, the incidence of lateralization was still higher when the malleus was absent.

Our perforation closure rate of 92.9% is consistent with reported lateral graft tympanoplasty success rates of 90%-100% (3,4,20,27,31,32). On average, the decrease in ABG was 6.82 ± 11.33 dB, somewhat lower than the 10-18 dB reported by others (3,4,32). Our patient sample included a significant subset that had previously undergone tympanoplasty (N = 45), and we found prior tympanoplasty was significantly associated with decreased improvement in ABG, similar to others (*P* < 0.05) (4,33). We also included ears with cholesteatoma, which were excluded by other analyses (31,32). There were also 12 ears (14.3%) left in discontinuity and 13 (15.5%) with ossicular fixation that opted to forgo a second, staged OCR or stapes surgery. Not surprisingly, these ears had less improvement in hearing compared with those that underwent OCR (*P* < 0.01).

## LIMITATIONS

This study is retrospective in nature and subject to the limitations inherent to chart reviews. Fifteen ears (15.2%) were excluded from the hearing analysis as they did not have postoperative audiograms. We tracked wound healing complications known to be associated with lateral graft tympanoplasties, acknowledging that other adverse events may have occurred and were not captured. There were no major systemic complications in the immediate postoperative period or reported by patients. Objective measures of the severity of diabetes (eg, hemoglobin A1c) or smoking (eg, packs per day) were also unavailable. It is possible that heavy smokers and poorly controlled diabetics are subject to healing complications not captured by this analysis. As tympanoplasty is primarily an elective procedure, it is possible that only patients with well-controlled diabetes were selected for surgery. Additionally, the subset of patients with active smoking histories (N = 11) and diabetes (N = 6) are relatively small, potentially preventing adequate power to detect a significant difference in outcomes.

## CONCLUSIONS

Lateral graft tympanoplasty is a reliable surgical technique, even in the presence of patient risk factors including active smoking, comorbid diabetes, and increasing age. Lateralization is more common when the malleus is absent. Further investigation with a larger, prospectively enrolled sample to collect objective metrics of comorbid disease severity is warranted to confirm our findings.

## FUNDING SOURCES

None declared.

## CONFLICT OF INTEREST

None declared.

## DATA AVAILABILITY STATEMENT

The datasets generated during and/or analyzed during the current study are not available.
